# Production of human vitronectin in *Nicotiana benthamiana* using the INPACT hyperexpression platform

**DOI:** 10.1111/pbi.12779

**Published:** 2017-07-20

**Authors:** Benjamin Dugdale, Maiko Kato, Pradeep Deo, Manuel Plan, Mark Harrison, Robyn Lloyd, Terry Walsh, Robert Harding, James Dale

**Affiliations:** ^1^ Centre for Tropical Crops and Biocommodities Queensland University of Technology (QUT) Brisbane QLD Australia; ^2^ Present address: Metabolomics Australia (UQ Node) Australian Institute for Bioengineering and Nanotechnology The University of Queensland St Lucia QLD 4072 Australia

**Keywords:** biopharming, INPACT, transgene expression, *Nicotiana benthamiana*, vitronectin, inducible

## Abstract

Human vitronectin (hVN) is a glycoprotein that functions as a cell adhesion molecule and a regulator of coagulation in blood plasma and the extracellular matrix. *In vitro*, hVN is added to serum‐free media in order to promote the adhesion of animal cells to tissue culture surfaces and the proliferation of undifferentiated stem cells. Here, we report the production of hVN in *Nicotiana benthamiana* using the inducible In Plant ACTivation (INPACT) hyperexpression platform. *N. benthamiana* plants were transformed with an INPACT expression cassette encoding hVN, and both the Tobacco yellow dwarf virus *Rep/RepA* activator and Tomato bushy stunt virus *p19* gene under the transcriptional control of the ethanol‐inducible AlcR*:alcA* gene switch. hVN expression was maximal 4–5 days postactivation of the INPACT platform with a dilute ethanol solution, and crude yields of the recombinant protein reached a maximum of 643 ± 78 mg/kg fresh weight. A three‐stage purification protocol was developed using heparin and polyhistidine tag affinity binding and size exclusion filtration, resulting in a plant‐made hVN product of >90% purity. Storage conditions for plant‐made hVN were identified that maximized the capacity of the recombinant protein to promote cell adhesion. Critically, plant‐made hVN was shown to be functionally equivalent to commercial, plasma‐derived hVN at promoting one‐half maximal attachment of murine fibroblast cells (BALB‐C/3T3) in serum‐free medium at <0.1 μg/cm^2^ to tissue culture plasticware. The INPACT platform represents an attractive means of producing large quantities of functional, animal‐free hVN for *in vitro* applications.

## Introduction

Human vitronectin (hVN), formerly known as serum spreading factor, is one of the major cell adhesion proteins found in the blood and the extracellular matrix (Conlan *et al*., 1988; Shaffer *et al*., 1984). Nascent hVN is converted to its mature form via cleavage of a 19‐amino acid secretion signal peptide at the N‐terminus. Circulating hVN occurs predominantly as a monomeric polypeptide (75 kDa) or as two polypeptides (65 and 10 kDa) linked by a disulphide bond (Tollefsen *et al*., 1990) and provides a regulatory link between a wide range of biological activities, including tissue repair, angiogenesis, haemostasis and metastasis (Schvartz *et al*., 1999). hVN interacts with diverse proteins via conserved regions located throughout the polypeptide chain (Jin and Varner, 2004; Preissner, 1991; Zhou *et al*., 2003) and the carboxyl‐terminal region contains a cryptic, arginine‐rich domain that binds heparin, an anticoagulant. This domain is only exposed after hVN binding to the thrombin–antithrombin III complex *in vivo* or denaturation with urea, heat, or acid *in vitro*, and it is this conformational change that activates self‐association of hVN into multimers with significantly increased affinity for heparin compared to the monomeric form (Preissner, 1991).

Vitronectin (VN) has been isolated from a range of mammalian sources and utilized for both research and clinical applications because of its adhesive properties. VN is most commonly used in animal cell culture to coat tissue culture surfaces in order to promote cell adhesion and induce cell spreading (Underwood and Bennett, 1989). Recombinant VN and its variants have also been used in combination with defined culture media to maintain pluripotency and growth of pluripotent stem cells (Li *et al*., 2010). Fusion of hVN to insulin‐like growth factor (IGF), IGF‐binding proteins and epidermal growth factor significantly enhances proliferation and migration of primary skin keratinocytes and the re‐epithelialization of wounds (Hollier *et al*., 2005; Upton *et al*., 2008; Xie *et al*., 2010). As a result, clinical hVN‐based treatments for improved wound management and healing have been developed, particularly where cell proliferation is required and/or wound repair has been delayed, such as in burns or ulcers (Upton *et al*., 2011). Further, hVN and its derivatives have been used to promote osseointegration of implantable devices by improving surface–cell interactions and increasing implant connectivity with surrounding bone (Cacchioli *et al*., 2009). hVN in its native form is traditionally purified from human blood plasma. As such, the protein must undergo strict regulatory testing for infectious agents and has become prohibitively expensive for applications in translational animal research.

Plant‐based protein production is a convenient means of manufacturing therapeutics and nontherapeutics normally isolated from animal sources, including blood plasma or tissues. Production in plants minimizes the risk of contamination with animal pathogens and obviates the need for expensive serological screening and more extensive purification. Such quality control processes are typically required for animal‐derived protein products destined for use in animal or human therapeutics. However, the economic feasibility of plant‐based protein production relative to conventional bacteria, yeast or insect cell bioreactor systems is fundamentally dependent upon recombinant protein yield. The use of plant viral vectors to amplify transgene copy number and virus‐derived gene products to suppress post‐transcriptional gene silencing (PTGS), an innate plant defence pathway that can specifically target transgene mRNA for degradation, has improved recombinant protein yields such that plants are becoming a competitive platform for the production of biologically equivalent proteins. Recent ‘deconstruction’ of the genomes of both DNA and RNA plant viruses has allowed the development of plant viral transgene expression vectors adapted for either short‐term, transient or long‐term, stable protein production in plants (reviewed in Lico *et al*., 2008 and Mortimer *et al*., 2015).

We recently described the In Plant ACTivation (INPACT) platform, an inducible, high‐level expression system for transgenic plants based upon the disaggregated DNA genome of a geminivirus, Tobacco yellow dwarf virus (TYDV) (Dugdale *et al*., 2013, 2014). The INPACT platform is unique in that the gene of interest is split and arranged such that its expression only occurs from extrachromosomal episomes that are released from the host chromosome in the presence of the TYDV‐encoded replication‐associated proteins, Rep and RepA. Temporal control of Rep/RepA expression is achieved using the ethanol‐responsive *alc* gene switch. Essentially, the INPACT platform provides the benefits of transient transgene expression in a stably transformed plant, thereby disconnecting plant growth from recombinant protein production. *Nicotiana benthamiana* transformed with an INPACT platform encoding hVN and activated with a dilute ethanol solution reached maximum crude yields of ~100 mg hVN/kg fresh weight (FW) (Dugdale *et al*., 2013). In the present study, we have integrated the gene encoding Tomato bushy stunt virus (TBSV) p19, a suppressor of PTGS, into the INPACT platform and describe its effects on recombinant hVN accumulation in *N. benthamiana*. The inclusion of TBSV p19 into the INPACT expression platform significantly increased maximum production of recombinant hVN by ~sixfold. A simple, three‐stage hVN purification process was developed, and the ability of plant‐made hVN to promote the attachment of murine fibroblast cells to tissue culture surfaces was compared to commercial hVN isolated from human plasma. The results demonstrate the potential of the INPACT expression platform for biopharming animal proteins *in planta*.

## Results

### Production of transgenic *N. benthamiana* plants containing an enhanced INPACT platform encoding hVN

**Figure 1:**
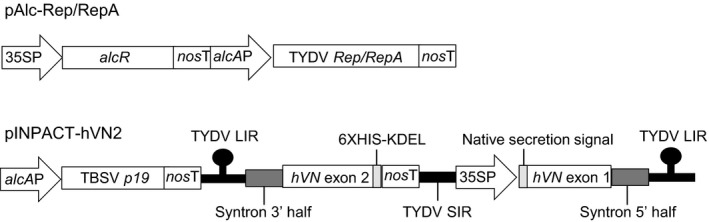
Schematic representation of the ethanol‐inducible Rep/RepA activator cassette (pAlc‐Rep/RepA) and the modified INPACT cassette encoding hVN and p19 (pINPACT‐hVN2). 35SP = CaMV 35S promoter, *nos*T = nopaline synthase gene terminator, *alc* *A*P = *alcA*:minimal CaMV 35S promoter fusion, *alcR* = gene encoding the alcohol receptor transcription factor, TYDV *Rep/RepA* = gene encoding the Tobacco yellow dwarf virus Rep/RepA activator proteins, TBSV *p19 *=* *gene encoding the Tomato bushy stunt virus 19 K protein, TYDV LIR = Tobacco yellow dwarf virus large intergenic region, syntron = synthetic intron, *hVN* exon = part of gene encoding the human vitronectin protein, TYDV SIR = Tobacco yellow dwarf virus small intergenic region, 6XHIS = polyhistidine affinity tag, KDEL = ER retention signal.

Synchronized activation of transgene amplification and expression from the INPACT platform is strongly dependent on the regulated expression of *Rep/RepA* activator genes. As such, the identification of elite *N. benthamiana* parent lines transformed with the ethanol‐inducible TYDV *Rep/RepA* cassette (pAlc‐Rep/RepA; Figure 1) was critical. Elite lines must satisfy two major criteria: (i) minimal *Rep/RepA* expression in the absence of the ethanol inducer molecule but rapid activation postethanol application, and (ii) minimal negative physiological impact of Rep/RepA accumulation on the plant, as overexpression of these gene products can be phytotoxic and cause rapid yellowing and necrosis (Dugdale *et al*., 2013). Transgenic *N. benthamiana* plants (NbAlc‐1, ‐2, ‐3, ‐4 and ‐5) containing the ethanol‐inducible TYDV *Rep/RepA* cassette were generated using *Agrobacterium tumefaciens*‐mediated leaf disc transformation, acclimatized in soil and activated with a 5% (v/v) ethanol foliar spray. Three days postethanol application, RNA was extracted from leaves and used as the template in a reverse transcription‐polymerase chain reaction (RT‐PCR) with primers specific for *Rep/RepA* gene sequences. *Rep/RepA* transcripts, indicated by an ~750‐bp RT‐PCR product, were detected in four of the five plants (NbAlc‐1, ‐2, ‐4 and ‐5) following ethanol application (Appendix S1). No PCR product was observed in RT‐PCRs without reverse transcriptase, indicating the absence of contaminating gDNA. RNA extracted from *N. tabacum* line NtSRN‐2 (a tobacco line containing the same pAlc‐Rep/RepA cassette) provided the positive control for the RT‐PCR. This tobacco line has been previously shown to express *Rep/RepA* by quantitative real‐time PCR (qRT‐PCR) following ethanol induction (Dugdale *et al*., 2013). Based upon the abundance of *Rep/RepA* transcripts in the RT‐PCR and the absence of an abnormal phenotype associated with Rep/RepA accumulation, NbAlc‐1 was selected as the elite line for supertransformation with the modified INPACT platform encoding hVN.

*Agrobacterium*‐mediated transformation was used to supertransform leaf discs from NbAlc‐1 with the modified INPACT platform encoding hVN and containing the TBSV *p19* gene under the transcriptional control of the *alcA* promoter (pINPACT‐hVN2, Figure 1). To facilitate accumulation of hVN, the native N‐terminal secretion signal was preserved and an ER retention signal (KDEL) added to the C‐terminus. For purification purposes, a C‐terminal polyhistidine affinity tag (6XHIS) was also included. Following cleavage of the 19‐amino acid secretion signal, plant‐made hVN has an approximate size of 469 amino acids and an estimated glycan‐free molecular weight of 53.63 kDa. Eleven independent *N. benthamiana* lines were regenerated on media containing both kanamycin and hygromycin.

### Identification of elite INPACT supertransformed lines

Detached leaves from the eleven transgenic *N. benthamiana* lines were excised and tested for ethanol‐induced accumulation of recombinant hVN. Leaves were incubated on MS0 solid media with small wells containing 5% (v/v) ethanol. Total soluble protein (TSP) was extracted 5 days postactivation and recombinant hVN levels determined by immunoblotting with an hVN‐specific monoclonal antibody. One line, T_0_‐2, was identified as a high hVN‐expressing INPACT plant (results not shown) and grown to maturity. Southern hybridization analysis using probes specific for either the *npt*II selection gene (within the pAlc‐Rep/RepA T‐DNA) or *hVN* gene (within the pINPACT‐hVN2 T‐DNA) showed this elite line contained a single integrated copy of both the pAlc‐Rep/RepA and pINPACT‐hVN2 cassettes (Appendix S2). Line T_0_‐2 was selfed, and the resulting 16 T_1_ generation events were screened for hVN accumulation using the same method as was used to analyse the T_0_ events. Three events expressing the highest levels of hVN (T_1_‐8, T_1_‐13 and T_1_‐15) were selected by immunoblotting (Appendix S3). These lines were selfed and three T_2_ generation events (T_2‐_1, T_2‐_2 and T_2‐_3) identified as high‐expressing hVN plants. All generations of transgenic plants developed normally and appeared phenotypically similar to wild‐type *N. benthamiana* plants in tissue culture and soil. Normal growth and development of transgenic plants was also observed in subsequent T_3_ generation plants grown in soil (Appendix S4).

### Ethanol‐activated expression of p19 and Rep/RepA in elite INPACT plants

**Figure 2:**
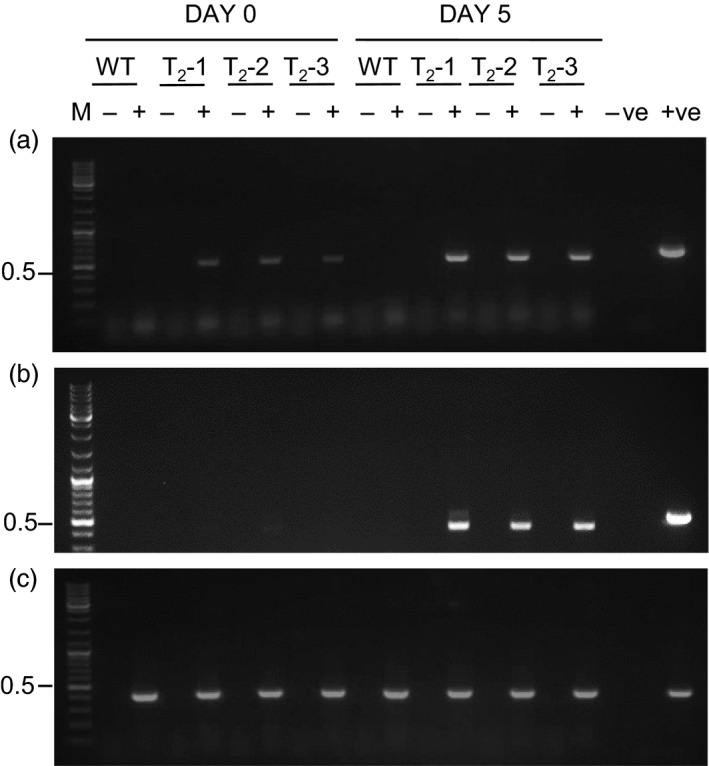
RT‐PCR to detect *p19* and *Rep/RepA* expression in high‐expressing INPACT lines following ethanol activation. Detached leaves of T_2_ generation INPACT lines (T_2_‐1, T_2_‐2, and T_2_‐3) were activated with a 0.5% (v/v) ethanol solution and sampled at 0 and 5 days postactivation. RNA was isolated from leaf samples and *p19* (top panel), *Rep/RepA* (middle panel) and actin housekeeping (bottom panel) gene expression detected by RT‐PCR. Amplified products were electrophoresed through a 1.5% agarose and stained with SYBR Safe DNA gel stain (Life Technologies). For PCR controls, water was used as the negative control (−ve) and 50 ng of plasmid DNA containing the *p19* gene (with syntron), TYDV *RepRepA* or actin genes were used as the positive controls (+ve). RT‐PCR without reverse transcriptase (−); RT‐PCR with reverse transcriptase (+); M = GeneRuler 1‐kb DNA ladder (Life Technologies); WT, wild type. Molecular weights marked in kb.

Detached leaves from T_2_ progeny plants, designated T_2‐_1, T_2‐_2 and T_2‐_3, were activated *in vitro* by incubation in liquid MS0 media containing 0.5% (v/v) ethanol for 5 days. RNA was extracted from leaves pre‐ (Day 0) and postethanol (Day 5) activation and analysed by RT‐PCR (Figure 2). *p19* transcripts (as indicated by a ~ 550‐bp RT‐PCR product) were detected in all transgenic progeny at Day 0, suggesting ‘leaky’ expression in the absence of the ethanol inducer molecule (Figure 2a). The relative abundance of these RT‐PCR products, however, increased by Day 5 suggesting that the addition of ethanol does increase *alcA*‐directed *p19* expression. In contrast, no *Rep/RepA* expression was observed prior to ethanol activation, and *Rep/RepA* transcripts were relatively abundant after activation (as indicated by a ~450‐bp RT‐PCR product; Figure 2b). The sizes of the RT‐PCR products for both *p19* and *Rep/RepA* were smaller than the PCR products amplified using plasmid DNA as templates, indicating correct processing of both the synthetic and TYDV Rep introns, respectively. No RT‐PCR products were observed in reactions using wild‐type *N. benthamiana* RNA as the template. RT‐PCR products (~400 bp) were obtained from all plant RNAs using primers designed to amplify the actin housekeeping gene (Figure 2c). No RT‐PCR products were observed in the absence of the reverse transcriptase enzyme.

### Kinetics of recombinant hVN accumulation and ethanol dose–response

**Figure 3:**
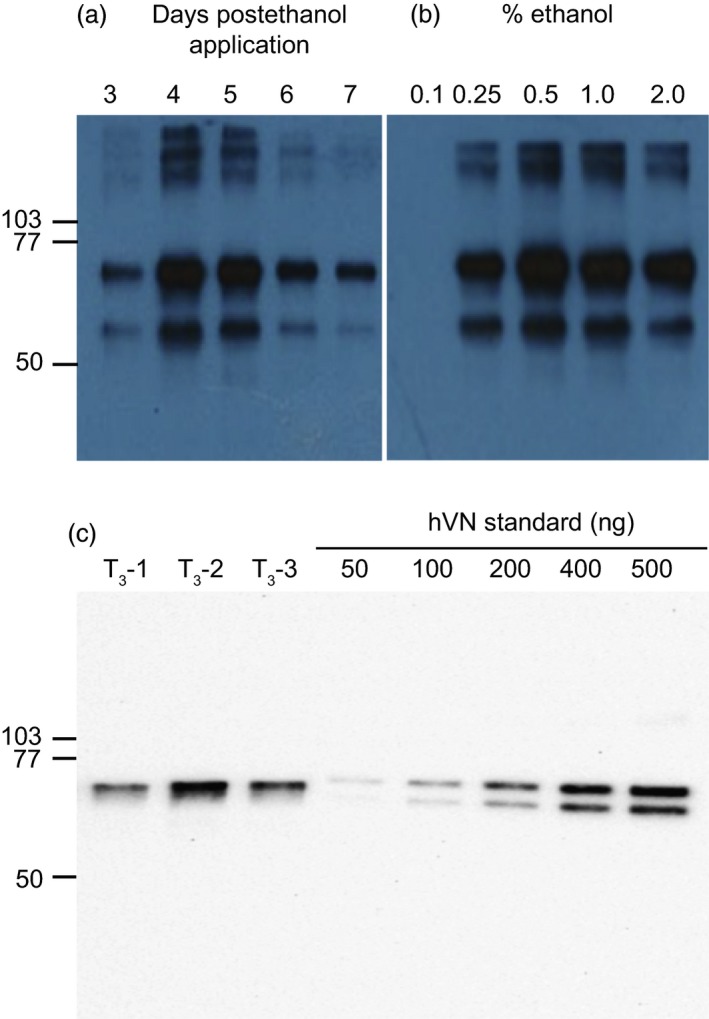
Time course and dose–response of ethanol activation for maximal INPACT‐based hVN expression and crude yield estimation. Detached leaves of elite T_0_ generation INPACT line, T_0_‐2, were activated in liquid MS0 media containing ethanol. For time‐course kinetics, leaves were incubated in 0.5% (v/v) ethanol solution and sampled 3, 4, 5, 6, and 7 days postactivation (Panel a). For dose–response, leaves of line T_0_‐2 were incubated in different concentrations (0.1, 0.25, 0.5, 1.0 and 2.0% v/v) of ethanol and sampled 5 days postactivation (Panel b). Samples in Panels a and b were extracted under nondenaturing conditions and TSP was normalized by Bradford protein assay. TSP (5 μg) was separated by SDS‐PAGE. For estimation of crude hVN yields in elite INPACT plants, detached leaves from three batches (marked T_3_‐1, T_3_‐2 and T_3_‐3) of T_3_ generation plantlets (eight plants from each T2 parental line) were incubated in 0.5% (v/v) ethanol solution and sampled 5 days postactivation (Panel c). TSP was extracted under denaturing conditions. TSP (5 μg) was separated by SDS‐PAGE. A standard curve of hVN (Promega) ranging from 50 to 500 ng was included for comparative yield estimates. Levels of hVN expression were determined by immunoblotting. Immunoblot signals were detected and measured using a ChemiDoc imaging system (Bio‐Rad). Molecular weights marked in kDa.

The kinetics of recombinant hVN accumulation were assessed by incubating transgenic leaf material from elite T_0_ generation line T_0_‐2 in liquid MS0 solution containing 0.5% (v/v) ethanol for 3, 4, 5, 6 and 7 days. hVN accumulation in leaf total soluble protein (TSP) extracts was measured by immunoblotting (Figure 3a). Maximum accumulation of the 75‐kDa form of recombinant hVN was observed 4–5 days after INPACT activation based on immunoblot signal intensities. Ethanol dose–response was assessed by incubating leaf material from the same plant in liquid MS0 solution containing increasing concentrations of ethanol (0.1%, 0.25%, 0.5%, 1.0% and 2.0% (v/v)). Five days after activation, hVN accumulation was measured in leaf TSP extracts by immunoblotting (Figure 3b). Maximum accumulation of the 75‐kDa form of recombinant hVN was observed using 0.5% (v/v) ethanol based on immunoblot signal intensity. Interestingly, high molecular weight hVN forms and an ~60 kDa hVN degradation product were also observed after immunoblotting. These extraneous hVN forms are likely multimers of hVN formed by self‐association under nondenaturing extraction conditions and a proteolytic cleavage product as a result of the freeze‐and‐thaw process prior to SDS‐PAGE and immunoblotting, respectively.

### Estimation of hVN crude yield

Yield estimates of plant‐made hVN were obtained using T_3_ generation plantlets. Seed from three independent T_2_ generation plants (T_2‐_1, T_2‐_2 and T_2‐_3) were sown onto MSO media containing both kanamycin and hygromycin antibiotics and 100% of the seed germinated within 1 week. This suggested all T_3_ generations plants contained both gene cassettes. Eight seedlings representing each T_2_ parent plant were randomly selected. Detached leaves from *in vitro* plants were activated for 5 days in liquid MS0 media containing 0.5% (v/v) ethanol. Leaf material from the eight seedlings was pooled (designated Batches T_3_‐1, T_3_‐2 and T_3_‐3) and hVN levels in the TSP compared to known quantities of commercial, pure hVN (Promega) by SDS‐PAGE and immunoblotting (Figure 3c). The use of denaturing buffer to extract plant‐made hVN and the immediate analysis of these extracts by SDS‐PAGE resulted in a single 75‐kDa band on the immunoblot. The commercial hVN standard appeared as a doublet because human‐derived hVN is clipped into two major products of 75 kDa and 65 kDa. A ChemiDoc imaging system (Bio‐Rad) was used to estimate the amount of hVN in TSP extracts by comparing signal intensity to the hVN standard curve ranging from 50 to 500 ng. This entire process was performed three times on separate occasions. The estimated hVN yields (expressed as mean ± standard error) from Batches T_3_‐1, T_3_‐2 and T_3_‐3 over three independent experiments were 577 ± 162, 709 ± 155, 642 ± 40 mg/kg (FW) of leaf, respectively (Table 1). Statistical analysis indicated there was no significant difference in yields between batches of T_3_ generation seedlings (*P* > 0.05), suggesting consistent activation and expression in this generation of plants. The overall estimated average hVN yield from T_3_ generation plants was 643 ± 78 mg/kg FW of leaf.

**Table 1:** Estimation of crude hVN yields in T_3_ generation INPACT plants

	Yield (mg hVN/kg FW leaf)			
T_3_ Generation plants	Experiment 1	Experiment 2	Experiment 3	Mean ± Standard Error
Batch T_3_‐1	370.9	973.5	385.6	577 ± 162
Batch T_3_‐2	874.0	923.2	330.8	709 ± 155
Batch T_3_‐3	581.6	740.1	605.2	642 ± 40
			Average overall yield	643 ± 78

### Purification of recombinant hVN from *N. benthamiana*

**Figure 4:**
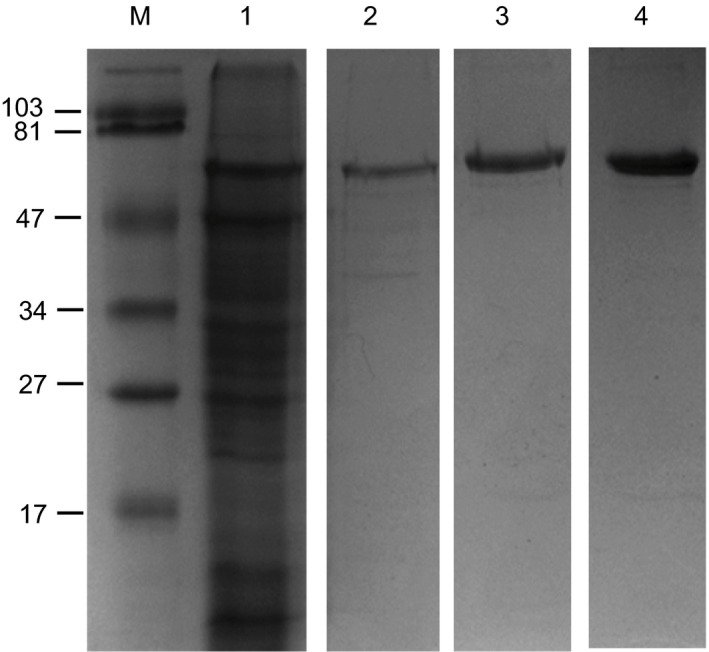
Purification of recombinant hVN from *N. benthamiana* using a three‐stage extraction protocol. Total leaf protein solubilized in 9 m urea (lane 1) was passed through a column containing Heparin Sepharose 6 Fast Flow resin and bound hVN eluted under high salt (500 mm NaCl) conditions (lane 2). HIS‐tagged hVN was then immobilized in TALON^®^ Superflow Metal Affinity Resin and eluted using imidazole (lane 3). Plant‐made hVN was further subjected to size exclusion filtration using an Amicon Ultra Centrifugal Filter (Ultracel‐30K) in order to remove low molecular weight protein contaminants and concentrate the product (lane 4). Protein samples representing each major step of the 3‐stage extraction protocol were electrophoresed through a 12% SDS‐polyacrylamide gel and stained with Coomassie Brilliant Blue R‐250. M = prestained SDS‐PAGE standards, low range (Bio‐Rad). Molecular weights marked in kDa.

Human vitronectin was purified from *N. benthamiana* using a three‐stage process based upon both affinity chromatography and size exclusion filtration. Samples from each key step (Figure 4) and all steps (Appendix S5) of the purification process were separated by SDS‐PAGE and visualized using Coomassie Blue dye. *N. benthamiana* leaf proteins were solubilized in 9 m urea to induce the conformational change in hVN that increases heparin affinity (Figure 4, lane 1). Denatured hVN was resolved from the majority of contaminating *N. benthamiana* leaf proteins by heparin affinity chromatography (Figure 4, lane 2). The purity of hVN after elution from the heparin affinity matrix was ~70%. hVN was further purified using metal affinity chromatography (Figure 4, lane 3). The purity of hVN after elution from the metal affinity matrix with 150 mm imidazole was ~80%–90%. hVN was separated from low molecular weight (<30 kDa) contaminants and concentrated to ~0.5 mg/mL using centrifugal ultrafiltration (Figure 4, lane 4). The purity of concentrated hVN was estimated to be >90% at a final yield of between 30 and 128 mg/kg FW of transgenic *N. benthamiana* leaf.

**Figure 5:**
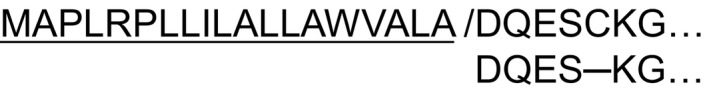
Comparison of the N‐terminal sequence of plant‐made hVN with native hVN. The native hVN 19 amino acid secretion signal is underlined and cleavage site marked (/). The blank amino acid read at position 5 of plant‐derived hVN is marked (─).

The identity of the purified protein from transgenic *N. benthamiana* leaf was confirmed by N‐terminal amino acid sequencing (Figure 5). No amino acid was detected on the fifth of seven cycles of Edman degradation. However, the presence of Cys‐S‐β‐propionamide (Cys‐S‐Pam), the product of cysteine alkylation by acrylamide under alkaline conditions, after the fifth Edman degradation cycle, suggested the presence of a cysteine residue. The resulting amino terminal sequence is identical to that predicted following cleavage of the native 19‐amino acid hVN secretion signal (Figure 5).

### Adhesive properties of plant‐made hVN

**Figure 6:**
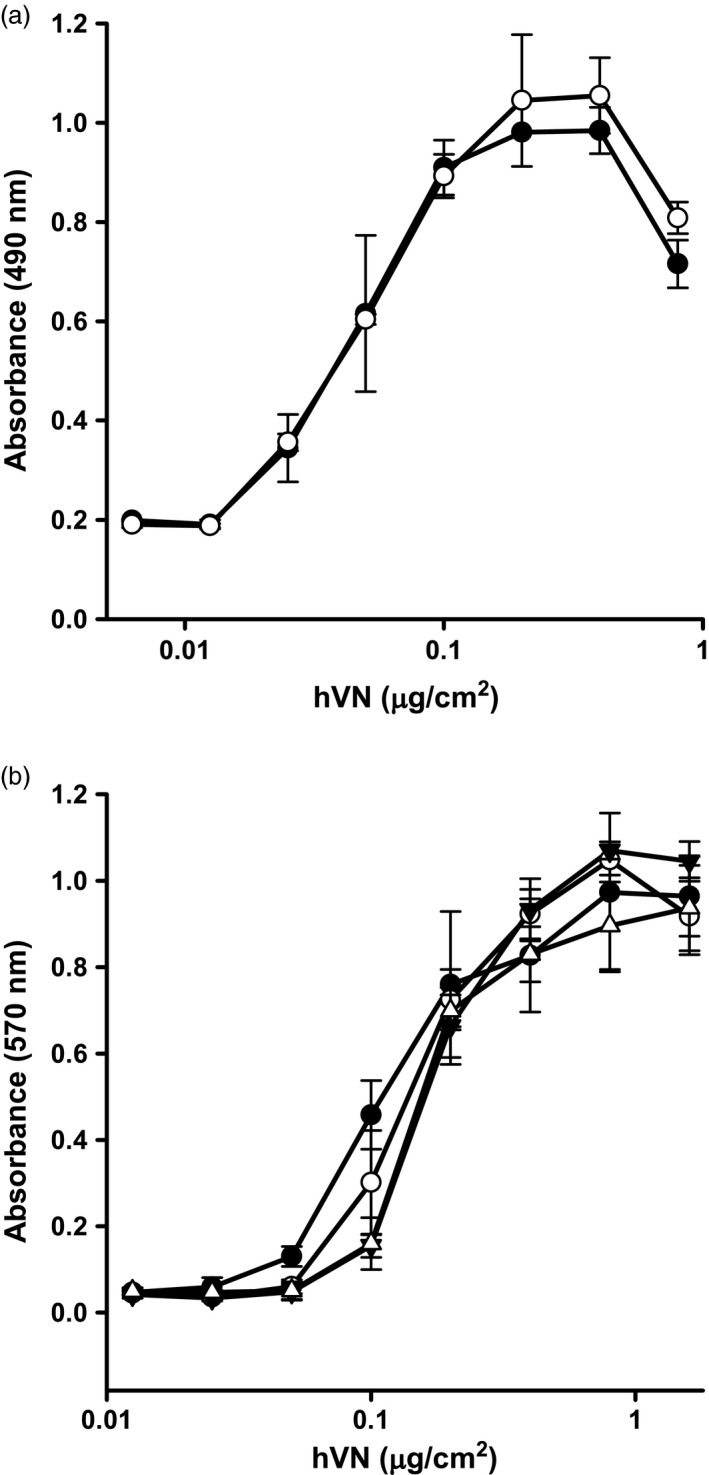
Adhesive properties of plant‐made hVN and effects of long‐term storage. Panel (a): The ability of plant‐made hVN (open circles) to promote the attachment of BALB‐C/3T3 cells in serum‐free medium to tissue culture plasticware was compared to commercial, plasma‐derived hVN (closed circles). MTS absorbance readings at 490 nm are directly proportional to the number of viable cells bound to the plasticware surface. Panel b: Plant‐made hVN was stored long term for 7 weeks as (i) a liquid at 4 °C (open triangles), (ii) frozen liquid at −80 °C (open circles) or (iii) lyophilized at −80 °C (closed triangles) and its ability to promote the attachment of BALB‐C/3T3 cells to tissue culture plasticware compared to commercial, plasma‐derived hVN (closed circles). MTT absorbance readings at 570 nm are directly proportional to the number of viable cells bound to the plasticware surface. Samples were analysed in triplicate, and error bars indicate mean ± standard deviation.

The capacity of plant‐made hVN to promote cell adhesion in tissue culture was assessed using murine fibroblast cells (BALB‐C/3T3) (Figure 6a). Commercial, plasma‐derived hVN (Promega) was used as a control. Fibroblast binding increased with increasing concentrations of both plant‐made hVN and commercial hVN. Maximum fibroblast adhesion (4 × 10^4^ cells/well) was observed at 0.4 μg hVN/cm^2^, and half‐maximum fibroblast adhesion occurred at ~0.05 μg hVN/cm^2^. The capacity of plant‐made hVN to promote fibroblast adhesion was statistically equivalent to that of the commercial product over all concentrations tested (*P* > 0.05).

Plant‐made, purified hVN was stored for 7 weeks as a liquid at 4 °C, a frozen liquid at −80 °C, and freeze dried powder at −80 °C to determine the effects of long‐term storage on protein activity. The capacity of plant‐made hVN to promote cell adhesion in tissue culture after storage was compared with commercial, plasma‐derived hVN (Promega) (Figure 6b). Maximum fibroblast adhesion for all hVN forms was observed at ~1.0 μg hVN/cm^2^. Half‐maximum fibroblast adhesion for stored plant‐derived hVN was ~0.2 μg hVN/cm^2^, compared to ~0.1 μg hVN/cm^2^ for the commercial control. The concentrations of both commercial and plant‐made hVN that promoted maximum and half‐maximum cell adhesion were significantly higher than was observed in Figure 6a, most likely because of variations in tetrazolium salt uptake by cells using MTT [3‐(4,5‐dimethylthiazol‐2‐yl)‐2,5‐diphenyltetrazolium bromide] versus MTS [3‐(4,5‐dimethylthiazol‐2‐yl)‐5‐(3‐carboxymethoxyphenyl)‐2‐(4‐sulfophenyl)‐2H‐tetrazolium]. Of the three conditions tested, storage of plant‐made hVN as a frozen liquid at −80 °C was the most effective at retaining adhesion promoting activity. Activity of hVN stored in this manner was statistically equivalent to the commercial product in the range of 0.2–1.6 μg/cm^2^.

## Discussion

Vitronectin is primarily used to promote cell attachment and proliferation *in vitro* but also has other therapeutic and nontherapeutic applications. These applications, however, are limited by the high cost of the native protein due to the extensive purification and serological testing required to ensure the absence of bloodborne pathogens. As such, *in planta* production of recombinant hVN that is functionally equivalent and free from animal pathogens by virtue of its source offers a cost‐effective alternative. We have previously demonstrated inducible production of recombinant hVN in transgenic *N. benthamiana* plants using the In Plant ACTivation (INPACT) platform (Dugdale *et al*., 2013). Soil‐acclimatized plants or detached leaves were activated with ethanol, and maximal hVN expression was detected by immunoblotting 4–5 days postapplication. Recombinant hVN was partially purified from leaves using heparin affinity chromatography, and crude yields were estimated at ~100 mg hVN/kg FW of leaf. In the present study, we have dramatically improved hVN yield by modification of the INPACT platform, developed an efficient recombinant hVN purification protocol, and demonstrated that recombinant hVN produced in transgenic *N. benthamiana* is functionally equivalent to commercial, human plasma‐derived VN.

The INPACT platform provides the benefits of high‐level, transient transgene expression in a stably transformed plant, whereby extrachromosomal copies of the INPACT transgene expression cassette are released, amplified, and transcribed only in the presence of ethanol and the Rep/RepA activator proteins. However, transient overexpression of heterologous genes can trigger PTGS, an innate plant pathogen defence mechanism that can significantly reduce steady state levels of recombinant protein accumulation (Voinnet, 2001; Voinnet *et al*., 1998; Waterhouse *et al*., 1998). In order to overcome this, virus‐encoded suppressors of PTGS can be co‐expressed with the gene of interest (Voinnet *et al*., 1999). One such suppressor, p19 from Tomato bushy stunt virus (TBSV), is known to sequester double‐stranded, small interfering RNA duplexes with high affinity, and co‐expression of this protein has been shown to significantly increase transient heterologous gene expression (Sainsbury and Lomonossoff, 2008; Sainsbury *et al*., 2009). However, p19 itself is phytotoxic and the generation of stably transformed plants expressing p19 is challenging (Scholthof, 2007). While a recombinant p19 mutant (R43W) that does not induce phytotoxicity has been developed (Saxena *et al*., 2011), this mutant form confers only a modest (1.7‐fold) increase in transgene expression levels. Therefore, in order to fully exploit the benefits of the TBSV p19 protein in transgenic plants, we integrated ethanol‐inducible expression of the TBSV *p19* gene into the INPACT platform for the production of hVN. We observed low level, ‘leaky’ *p19* transcription in the absence of ethanol induction but there was no evidence of a negative impact upon phenotypic parameters such as plant growth or development in the T_0_ to T_3_ generation events. Further, we demonstrated that co‐expression of *p19* and *hVN* increased recombinant hVN accumulation up to sixfold, relative to the unmodified INPACT platform. This suggests that PTGS is likely a limiting factor for INPACT‐based recombinant protein expression, but it can be overcome by inducible expression of a PTGS suppressor gene.

Intracellular targeting of recombinant proteins can significantly influence the steady state levels to which they accumulate *in planta* (Harrison *et al*., 2011; Streatfield *et al*., 2003). We previously tested whether localized accumulation of recombinant hVN in intracellular compartments greatly influenced its yield, by targeting the protein to the cytoplasm, endoplasmic reticulum (ER), apoplast, mitochondria or chloroplast. We found that hVN retention in the ER resulted in maximum accumulation in *N. tabacum* leaves (results not shown). Native hVN is glycosylated at three sites which accounts for as much as 30% of the mass of the mature protein (Schvartz *et al*., 1999), and changes in hVN glycosylation alter its ability to form multimers and bind collagen (Sano *et al*., 2007). However, glycosylation does not appear essential for some of its biological activities as glycan removal can increase collagen binding (Sano *et al*., 2007) and bacteria‐made VN has been used in chemically defined animal cell culture systems (Chen *et al*., 2011). In the present study, the native hVN amino‐terminal secretion signal was preserved so that the recombinant protein would enter the ER and an ER retention signal (KDEL) added to the carboxyl‐terminus of hVN to enhance accumulation.

We have developed a small‐scale production system using leaves harvested from transgenic plants maintained *in vitro* and activated with ethanol in solution. Elite T_0_ generation *N. benthamiana* INPACT events expressing high levels of recombinant hVN were micro‐propagated in tissue culture using a rapid axillary shoot induction method (Deo *et al*., 2015) that allowed generation of ~200 individual plants. We routinely produced ~150 g of *N. benthamiana* leaf mass per week using a rotating subculture system and accumulated >3 kg of leaves in a 5‐month period. Detached leaves were activated in tissue culture containing a liquid growth media supplemented with 0.5% (v/v) ethanol and harvested for purification after 5 days when hVN accumulation was at its peak. Activated leaves could be stored at −80 °C or used immediately for hVN extraction.

Laboratory‐scale purification of recombinant hVN was routinely undertaken using 10–30 g of *N. benthamiana* leaves, but we have successfully scaled up purification to 100 g quantities of leaves in the present study. Interestingly, higher plants encode hVN analogues that function in plasma membrane–cell wall adhesion (Zhu *et al*., 1993), bacterium–plant interaction (Wagner and Matthysse, 1992) and pollen tube extension (Sanders *et al*., 1991). One such protein, tobacco PVN1 (plant vitronectin‐like 1), is predominantly localized to the cell wall and has been shown to bind both glass and heparin (Zhu *et al*., 1994). *N. benthamiana* encodes a protein with 97% similarity to tobacco PVN1 (Nakasugi *et al*., 2013) and contains an RYD motif with functional similarity to the hVN RGD binding domain. Therefore, a second affinity chromatography step (*i.e*. immobilized metal) was included in our hVN purification process to minimize the likelihood of contamination with *N. benthamiana* PVN1. We observed that recombinant hVN yield was significantly affected by the age and physiological status of plants *in vitro*. Leaves obtained from freshly propagated *N. benthamiana in vitro* plants were generally larger, appeared healthier and yielded the maximum level of >90% pure recombinant hVN (128 mg/kg FW). In contrast, leaves from the third harvest and beyond were generally smaller, more chlorotic and had reduced hVN content (30 mg/kg FW).

Optimization of the molecular features of the INPACT platform resulted in a sixfold increase in recombinant hVN accumulation compared to the unmodified INPACT platform, and the development of an efficient extraction protocol produced a plant‐made hVN of >90% estimated purity that was functionally equivalent to the native human protein. While transient agroinfiltration‐based expression remains the preferred method for rapid recombinant protein production in plants, there is still an obvious need for extended, large‐scale production capacities. The elite transgenic INPACT plants and the seed bank generated in this study represent a permanent genetic resource for the consistent and high‐level production of this valuable protein into the future.

## Materials and methods

### Vector construction

The CaMV 35S promoter (35SP) controlling expression of the *hygromycin B phosphotransferase* (*hph*) selection gene in pCAMBIA1300 was replaced with the *nopaline synthase* gene promoter (*nos*P). The *nos*P and *hph* genes were amplified by PCR and fused using overlapping PCR with the following primer pairs; hph‐F (5′‐TCTCCGCTCATGATCATGAAAAAGCCTGAACTCACCGCGA‐3′) and hph‐R (5′‐CTCGAGCTTGTCGATCGACAGATCCGGTCGGCATC‐3′), and nosP‐F (5′‐GAATTCTCTAGACACGTGAGATCCGGTGCAGATTATTTGGATTGAGAGTG‐3′) and nosP‐R (5′‐TTCAGGCTTTTTCATGATCATGAGCGGAGAATTAAGGGAG‐3′). The resulting *nos*P‐*hph* fusion was ligated into pCAMBIA1300 using *Xho*I and *Eco*RI restriction sites. An INPACT expression cassette encoding the GUS reporter gene was excised from pINPACT‐GUS (Dugdale *et al*., 2013) and ligated into the above vector using *Eco*RI/*Hind*III restriction sites. The nearly complete INPACT cassette encoding hVN (with native secretion signal, KDEL retention signal and polyhistidine affinity tag) was then excised from pINPACT‐hVN (Dugdale *et al*., 2013) and ligated into the vector using *Swa*I/*Pac*I restriction sites to create pINPACT‐hVN‐nos. Wild‐type TBSV *p19* (GenBank Accession M21958.1) was codon modified to include human and *N. tabacum* first preferred codons and an 84‐bp synthetic intron (syntron; Dugdale *et al*., 2013) between the AG/GT at nucleotide position 201. The modified *p19* gene was chemically synthesized by GeneArt^®^ (Life Technologies, Mount Waverley, VIC, Australia) and ligated upstream of the *nopaline synthase* gene terminator (*nos*T) in the plasmid pACN2 using unique *Pst*I restriction sites. The final INPACT hVN expression vector was constructed by three‐way ligation of the following fragments: *Pml*l/*Xba*I digested pINPACT‐hVN‐nos backbone, *Pml*I/*Bam*HI digested *alcA* promoter sequence and *Bam*HI/*Xba*I digested *p19*‐nosT sequence from pACN2. The resulting vector was designated pINPACT‐hVN2 (Figure 1).

Construction of the vector pAlc‐Rep/RepA, a pBIN‐based vector backbone containing (i) the TYDV *Rep/RepA* activator genes downstream of the *alcA* promoter, (ii) the *alcR* transcription factor gene under the transcriptional control of 35SP and (iii) the *neomycin phosphotransferase* (*nptII*) resistance gene for kanamycin selection of transformed plant cells, has been previously described (Figure 1; Dugdale *et al*., 2013).

### Stable transformation of *N. benthamiana*

All vectors for stable transformation were mobilized into *Agrobacterium tumefaciens* (strain LBA4404) by electroporation. *A. tumefaciens*‐mediated transformation of *N. benthamiana* leaf discs and their regeneration were as described by Horsch *et al*. (1985). Transgenic plants containing the pAlc‐Rep/RepA cassette were selected and regenerated in media containing kanamycin (200 μg/mL). Ethanol‐inducible expression of the *Rep/RepA* genes in these lines was assessed by RT‐PCR. Leaves from event NbAlc‐1 were subsequently used for supertransformation with recombinant *A. tumefaciens* harbouring pINPACT‐hVN2. To ensure supertransformed plants contained both pAlc‐Rep/RepA and pINPACT‐hVN2 expression cassettes and were independent events, plantlets were excised from different leaf pieces and regenerated in media containing both kanamycin (200 μg/mL) and hygromycin (25 μg/mL). *In vitro* or soil‐acclimatized plants were maintained in a controlled environment chamber with a 16‐h photoperiod at 25 °C and grown to the 8‐ to 10‐leaf stage prior to harvest and ethanol activation.

### Reverse transcription PCR (RT‐PCR)

Leaf samples were immediately snap‐frozen in liquid nitrogen following harvesting. Tri reagent (Sigma‐Aldrich, Castle Hill, NSW, Australia) was used to extract total RNA from tissue according to the manufacturer's instructions and the method of Azevedo *et al*. (2003). Oligo (dT) 18 primer was used to synthesize first‐strand complementary DNA from total RNA using M‐MLV Reverse Transcriptase (Promega, Alexandria, NSW, Australia) according to the manufacturer's instructions. Reactions were also prepared without reverse transcriptase to confirm the absence of contaminating gDNA. PCRs were performed using GoTaq Green master mix (Promega) and the following cycling conditions 94 °C for 5 min followed by 29 cycles of 94 °C for 30 s, 55 °C for 30 s, 72 °C for 30 s, and 72 °C for 10 min. Primer sets were as follows; TYDV *Rep*/*RepA*–Rep/RepA‐F (5′‐TCAGACTGGCAACCTATT‐3′) and Rep/RepA‐R (5′‐GCGAACTATTATCCAGAC‐3′), wild‐type TBSV *p19*–P19‐F (5′‐CCATGGAAAGGGCTATTCAGGGAAATGATG‐3′) and P19‐R (5′‐GAGCTCTCACTCGGATTCTTTCTCAAAGTC‐3′), and actin–Actin‐F (5′‐CTATTCTCCGCTTTGGACTTGGCA‐3′) and Actin‐R (5′‐AGGACCTCAGGACAACGGAAACG‐3′).

### Ethanol activation

The INPACT platform was activated in whole or detached leaves using three different methods. For soil‐acclimatized plants, 5% (v/v) ethanol in water was applied as a foliar spray and root drench. For rapid screening of transgenic lines, leaves of *in vitro N. benthamiana* plants were ethanol activated in sealed Petri dishes. Leaves were placed adaxial side down onto solid MS0 media containing a 5‐mm‐wide and 5‐mm‐deep well filled with 3 mL of 5% (v/v) ethanol. For time‐course kinetics, dose–response, yield estimates and laboratory‐scale hVN purification, leaves from tissue culture *N. benthamiana* plants were harvested, placed in a sterile 500‐mL tissue culture vessel, and immersed in 150 mL of MS0 media (Murashige and Skoog, 1962) containing 0.5% (v/v) ethanol. The vessel was agitated on a flatbed rotary shaker (60 rpm) for 5 days with a 16‐h photoperiod at 25 °C. For time‐course kinetics, sampling time was varied (3, 4, 5, 6, and 7 days postactivation), and for dose–response, ethanol concentration was varied (0.1%, 0.25%, 0.5%, 1.0%, and 2.0% (v/v)). Excess liquid was removed prior to extraction or freezing in liquid nitrogen and storage at −80 °C.

### Purification of hVN from *N. benthamiana* leaves

Leaves were ground to a powder in liquid nitrogen using a mortar and pestle. For rapid hVN detection, TSP was extracted in five volumes of either 200 mm phosphate buffer (pH 7) or extraction buffer (9 m urea, 50 mm phosphate, 10 mm β‐mercaptoethanol, pH 7) and separated by SDS‐PAGE for immunoblotting. For laboratory‐scale purification, extraction buffer containing EDTA‐free Complete Protease Cocktail Inhibitor (Roche, Castle Hill, NSW, Australia) was added to the leaf powder at a ratio of 7.5 mL per gram FW. The concentration of urea was increased from 8 m (that used to isolate hVN from blood plasma (Yatohgo *et al*., 1988)), to 9 m in order to compensate for high leaf water content. The resulting slurry was agitated at 15 rpm on an orbital wheel shaker for 15–30 min at room temperature. Larger plant debris was removed by filtration through Miracloth (VWR, Murarrie, QLD, Australia) prior to clarification by centrifugation at 20 000* **g*** for 20 min at 20 °C. A Heparin Sepharose 6 Fast Flow (GE Healthcare, Mansfield, QLD, Australia) column was prepared with a packed bed volume equivalent to 1 mL/5 g starting leaf material and equilibrated with three column volumes of HS buffer (8 m urea, 50 mm phosphate, pH 7). The supernatant was loaded directly onto the equilibrated Heparin Sepharose. The Heparin Sepharose was washed with five column volumes of HS buffer and bound hVN eluted using five column volumes of HS Elution buffer (HS Column buffer supplemented with 500 mm NaCl).

A column of TALON^®^ Superflow Metal Affinity Resin (Sigma) was prepared with a packed bed volume equivalent to 1 mL/5 g leaf material and equilibrated with three column volumes of HS Elution buffer. The eluent from the Heparin Sepharose column was loaded directly onto the equilibrated TALON® column. The column was washed with five column volumes of HS Elution buffer and bound HIS‐tagged hVN eluted using five column volumes of TALON Elution buffer (HS Elution buffer supplemented with 150 mm imidazole).

An Amicon Ultra Centrifugal Filter unit (Ultracel‐10K, Millipore) was equilibrated using 3 mL of HS Elution buffer and centrifugation at 5000 ***g*** for 5 min at 18 °C. The eluent from the TALON^®^ column was diluted 1 : 2 with HS Elution buffer (to decrease the imidazole concentration to 75 mm) then loaded onto the Ultracel‐10K filter and centrifuged at 5000 ***g*** for 10 min at 18 °C. The filter was washed five to 6 times with 3–4 mL of HS Elution buffer and centrifuged at 5000 ***g*** for 10 min at 18 °C until the final retained volume was between 100 and 500 μL.

A Puradisc FP 30 PTFE, 0.2‐μm sterile syringe filter (Thermo Fisher, Scoresby, VIC, Australia) was equilibrated with 1 mL of HS elution buffer. hVN retained after size exclusion filtration was sterilized using the equilibrated filter. Purified plant‐made hVN was either stored short‐term at 4 °C or long term at −80 °C with or without freeze drying.

Samples (5 μL) from each step of the purification protocol were collected and stored on ice prior to PAGE analysis. Purified plant‐made hVN was quantified using the Bradford Protein Assay microtitre plate procedure (Bio‐Rad, Regents Park, NSW, Australia) according to the manufacturer's instructions and known amounts of commercial purified hVN (Promega). Absorbance at 595 nm was determined using a Beckman Coulter™ AD200 plate reader, and samples were analysed in triplicate.

### PAGE, immunoblotting, N‐terminus sequencing and yield estimation

PAGE and immunoblotting for detection of hVN was carried out as described by Dugdale *et al*. (2013). For amino‐terminal sequencing, 3 μg of plant‐made hVN was subjected to SDS‐PAGE, transferred to PVDF membrane and stained with Ponceau dye. The major 75‐kDa band was excised from the PVDF membrane, and the first seven amino‐terminal residues were sequenced using an Applied Biosystems 494 Precise Protein Sequencing System (Australian Proteome Analysis Facility, Macquarie University, NSW). For yield estimation, Clarity Western ECL substrate (Bio‐Rad) was formulated according to the manufacturer and applied as a 1 : 10 dilution to the membrane. Signal strength was detected using a ChemiDoc imaging system (Bio‐Rad) and yield calculated from a hVN standard curve ranging from 50 to 500 ng. Average plant‐made hVN yield is presented as mean ± standard error.

### Cell adhesion assay

Plant‐made and plasma‐derived hVN (Promega) were diluted in Dulbecco's PBS (DPBS, Life Technologies) to concentrations ranging between 1.6, 0.800, 0.400, 0.200, 0.100, 0.050, 0.02500, 0.0125 and 0 μg/cm^2^ in 100 μL. Wells of a Nunc MaxiSorp® flat‐bottom 96‐well plate (Sigma) were coated with 100 μL of each hVN dilution in triplicate. Plates were incubated for 2 h at room temperature then rinsed 3 times with 250 μL of DPBS per well. After rinsing, 200 μL of DPBS blocking solution (DPBS with 2 mg/mL bovine serum albumin (BSA)) was added to each well and the plate was incubated for 1 h at room temperature. The BSA blocking solution was removed prior to adding animal cells. Three‐day‐old BALB‐C/3T3 cells were harvested by trypsinization and pelleted by centrifugation at 200 ***g*** for 5 min at room temperature. Cells were resuspended in 1 mL Dulbecco's Modified Eagle Medium (DMEM) containing 10% (v/v) foetal bovine serum (Life Technologies). Viable cell counts were estimated using trypan blue exclusion dye (Life Technologies) and cell concentrations adjusted to 4 × 10^5^ cells/mL. An aliquot (100 μL) of cell suspension (4 × 10^5^ cells/mL) was added to each hVN‐coated well, and the plate was incubated at 37 °C with 5% (v/v) CO_2_ for 1 h. Unattached cells were gently aspirated from the wells using a multichannel pipette and the attached cells gently washed 3 times with 250 μL of serum‐free DMEM (Sigma) per well. Cell densities were measured using either MTS or MTT substrate methodologies. For MTS substrate, 100 μL of serum‐free DMEM and 20 μL of CellTiter 96® AQueous One Solution Cell Proliferation Assay reagent (Promega) was added to each well and incubated at 37 °C with 5% (v/v) CO_2_ for 1 h. Absorbance at 490 nm (compared to absorbance at a reference wavelength of 690 nm) was measured using a Beckman Coulter™ plate reader and data averaged using three replicates. Mean absorbance at 490 nm versus the hVN concentration was plotted in order to determine ED_50_. For the MTT substrate, 10 μL of MTT (5 mg/mL) in DPBS was added to each well and incubated at 37 °C with 5% (v/v) CO_2_ for 3–4 h until a dark precipitate formed. The media were then aspirated and 200 μL of 100% (w/v) DMSO added to each well in order to solubilize the precipitate. Absorbance was measured at 570 nm (compared to an absorbance at a reference wavelength of 630 nm) and plotted against hVN concentration to determine ED_50_, as above.

### Statistical analysis

One‐way analysis of variance was performed to compare different batch yields or different cell binding activities (*P* ˂ 0.05 was considered significant). Data values were expressed as mean± standard error for yield estimates and mean ± standard deviation for cell attachment assays.

## Conflict of Interest

Authors declare no conflict of interest.

## Supporting information


**Appendix S1** Reverse Transcription (RT)‐PCR to detect *Rep/RepA* expression in transgenic *N. benthamiana* parent lines following ethanol activation.

**Appendix S2** Southern hybridization to determine copy number.

**Appendix S3** Identification of high hVN‐expressing T_1_ generation INPACT lines.

**Appendix S4** Growth and development of transgenic plants in soil.

**Appendix S5** Purification of recombinant hVN from *N. benthamiana* using a three‐ stage extraction protocol.
